# Hospital and Institutional News

**Published:** 1920-06-12

**Authors:** 


### HOSPITAL AND INSTITUTIONAL NEWS.

### BIRTHDAY HONOURS.

The names of medical men do not figure largely in the Honours Lists published on June 3 and 5. The former list, indeed, contains none. In the latter are the following: Professor F. W. Andrewes, M.D., F.R.S., Pathologist to St. Bartholomew's Hospital, who receives a knighthood for his valuable work in medical research; the same honour is conferred on Alderman Charles O'Brien, M.R.C.S., L.R.C.P., Mayor of Eastbourne in 1903 and from 1916 to 1919; and in the Indian list on Colonel Hormasjee Eduljee Banatvala, I.M.S. (retired), late Inspector-General Civil Hospitals, Assam. A Knight Commandership of the British Empire (Civil Division) has been conferred on Mr. Jas. Dundas-Grant, M.D., F.R.C.S., the eminent aural specialist; on Mr. Arnold Lawson, M.D., F.R.C.S., the well-known ophthalmic surgeon, for his medical and surgical work at St. Dunstan's Hostel for the Blind; and on Colonel Wm. Taylor, C.B., M.D., ex-President of the Royal College of Surgeons in Ireland. The Kaisar-i-Hind Medal, 1st Class, has been awarded to Dhanjibhai Hormasji Mehta, medical officer, Patao Hospital, Baroda; and to Shamrao Ramrao Moolgavkar, Esq., Principal Medical Officer, Bikaner State, Rajputana. This medal has also been awarded to Miss Millicent Vere Webb, lady superintendent, Dufferin Victoria Hospital, Calcutta, Bengal.

### LORD DAWSON'S SCHEME.

On page 273 of this issue we publish some impressions which the outline of this scheme have made upon a valued contributor to this Journal, and one whose experience in dealing with the health problems of some of the most representative parts of the country has extended over many years. It is possible that his fears of an ultimate rigid and complete State control of medicine are not fully justified—upon present evidence, at least—but we heartily agree with his whole-hearted criticism of any attempt at over-organisation in the Great Britain of to-day. Also, his appreciation of the essential place of the voluntary hospital system is a pleasing confirmation of the views we have ourselves already expressed on this subject, and is the more valuable when coming from the pen of a prominent member of the existing Public Health Service.

### £5,000 FOR SUGGESTIONS.

The subject of possible new adaptations of rubber to the routine work of hospitals is one which should prove attractive to many of our readers, more particularly as the Rubber Growers' Association have decided to offer £5,000 as prizes in the form of a competition for ideas and suggestions for extending the present applications of rubber. Medical work and medical workers in general touch upon but a small part of the possible spheres of usefulness of this product, but the potential value of any distinct advance in hospital technique is so great that we think the subject should be of no little interest to some of those who scan these pages. All inquiries should be sent to the Rubber Growers' Association (Dept. C.), 38 Eastcheap, London, E.C. 3, and we would advise that request be made there for the descriptive literature of the competition before any suggestions are actually forwarded.

### THE LONDON HOSPITAL'S APPEAL.

As we go to press the amount received in response to Lord Knutsford's letter in *The Times* is £30,000. Included in this is the National Sunday League's gift of 500 guineas to keep the hospital for one day. The day selected by the League was last Sunday, when the whole cost of running the hospital was defrayed by it. What the London Hospital wants is new income of £80,000 a year, which is the amount by which the present total income falls short of present-day requirements.

### CLOSING OF THE NATIONAL HOSPITAL.

In view of the serious financial position of the National Hospital for the Paralysed and Epileptic, the Board of Management has been obliged to close the in-patient department, and has directed that no further patients are to be admitted after the end of the current week. The hospital is quite full, and the cases will be evacuated as soon as the nature of the cases permits. Notice has been sent to the 144 patients on the waiting-list that they cannot be taken in. The work in the out-patient department, the country branch at East Finchley, the branches supported by the Ministry of Pensions, and the massage school and hostel will continue as usual.

### WESTMINSTER HOSPITAL.

We are informed that there is no foundation for the report that the site of Westminster Hospital has been sold.

### IRELAND AND THE VOLUNTARY SYSTEM.

The financial position of the Dublin Hospitals has led to an energetic attempt to raise by a collective appeal the £100,000 which is at once needed to pay their debts. Luckily the Appeal Committee, of which Viscount Powerscourt is President, appears to consist of men who believe in the voluntary principle, and the speeches delivered at the big meeting lately held in the theatre of the Royal Dublin Society, Kildare Street, were refreshingly free from doubt or timidity. Those in charge of the appeal, who are known as the council, include four representatives nominated by each hospital, one of each religious body, four of Labour, four of the Dublin Corporation, four ex-Service men, and, briefly, the most active public men in Dublin. Englishmen must remember that £100,000 is at least as alarming to Irishmen as one million would be here, and, in consequence, it is suggested that since all Ireland uses the Dublin hospitals, the appeal should be made broadcast. So long as the organisation is broadcast also, and a general appeal does not stop short at a general circular, we see little objection to the plan. The interests of local hospitals need not suffer, and, as our own King Edward's Hospital Fund has shown, a central fund may even benefit individual hospitals. We speak of this Dublin Appeal as a central fund, because, if the organisation is worthy of the cause, Lord Powerscourt and the Presidents of the Irish Royal Colleges should insist that the organisation does not lapse when once the immediate appeal is over. The Appeal Council can easily become later a maintenance council. That is the secondary end to keep in mind.

### THE LESSON OF "THE CRISIS."

It is always amusing, if not very safe, to prophesy. We think there is much to be said for those who believe that the big hospitals here will eventually adopt the American plan of having for their chief administrators medical superintendents. If so, finance should still be left to the layman, who after all has often qualified as carefully in *this* branch as any doctor in therapeutics. Wise men take long views, and at present the lay superintendents, so busy with appeals, should do their best to develop an organisation which can be adopted after the course of the appeal itself shall have been run. There is no reason, in the nature of things, why a donor, who will respond in an emergency, should not be convinced that the emergency need not arise if he will spread his gifts a little, and make a quiet habit of that which has hitherto been a general impulse only. The stray donor is too much neglected, too little nursed, though it is the boast of some secretaries that no one who writes or visits their hospital is ever forgotten by it afterwards. Yet the hospital secretaries who make this boast do not possess lists of annual subscribers conspicuously larger than those of other institutions. Now is the time to act, not only because the public is in the mood to give, but also to create a department which will be essential to the hospital if and when the medical superintendent is appointed over it.

### THE CARDIFF APPEAL.

A sum of some £120,000 has been raised so far in response to the appeal for King Edward VII. Hospital, Cardiff. This sum of course is needed for capital purposes and the reduction of debt. But it is very satisfactory to note that together with the appeal efforts are being made successfully to increase the income from annual subscriptions. An admirable example has been set by the Barry Graving Dock and Engineering Company, Limited, which has increased its annual subscription from five guineas to £100. Whether this is a personal triumph for Mr. Leonard Rea's persuasiveness we do not know, but it shows a sense of public spirit and goodwill on the part of the company which ought to be infectious. A standard of duty has now been set, and we fancy that the Barry Dock Company will not willingly remain in a splendid isolation, but will prefer that other companies shall also do their duty to King Edward VII. Hospital. Here again the ultimate success of this appeal, which unfaltering endeavour can make certain, should leave behind it the skeleton at least of a maintenance fund, so as to convert as many of the donors as possible into annual subscribers. This can be done especially in the provinces, where there is an *esprit de corps* which is the envy of London.

### SIR LAMBERT ORMSBY ON THE NURSING OF CHILDREN.

As senior medical officer of the National Children's Hospital, Dublin, Sir Lambert Ormsby lately mentioned the concern aroused by the resolution of the Dublin Corporation to withdraw grants from those hospitals which refused to reduce the nursing hours to eight per day. This, he said, placed the nurse on a par with the artisan or labourer, who was supposed to work continuously for the eight hours of his daily shift. But nurses, whether at private cases or in hospital wards, did not usually work continuously. Half her time, Sir Lambert added, she might be reading or sewing or writing letters, though she must be ready to answer a bell or call at any moment. If the rule suggested by the Corporation was carried out, it would cost the hospitals which adopted it in extra nurses more than the value of the Corporation's grant! In children's hospitals, he went on, the greater part of the work is easy, and nurses and probationers are allowed out of hospital every other day from 3 p.m. to ten o'clock at night. Apart from the fact that a weekly system of hours can get over most of the difficulties of the *eight-hour day*, will our readers agree that the nursing of children is mostly easy?

### A COMMITTEEMAN'S CONFESSION.

A hospital committeeman writes: "Properly organised, I am confident that each hospital can meet its debts and carry on satisfactorily in the future, but it is my experience, and I am on the committee of ——— hospital, that they must be conducted upon more strictly business lines than is the case at present. Committees must welcome more outside voluntary help than they often do at present. Many managers of hospitals are chosen for their position, or because they have been decorated, whereas the chief qualifications should be business knowledge, hospital experience, and a lively affection for the hospital to which they are attached." Except that our correspondent places the qualifications of a hospital committeeman in exactly the opposite order to that which we should choose, we cannot quarrel with his criticism. Hospitals are not merely businesses: they are centres for disinterested work. Hospital life, like nursing, is not merely a profession: it is a vocation. In the absence of a sense of that vocation all other qualifications are dangerous.

### HIGHER SICK BENEFIT.

In a letter to the St. Pancras Council, Dr. Addison, dealing with the subject of sick benefit under the Insurance Act in relation to a possible epidemic of influenza, states that the date from which benefit is payable was thoroughly considered in 1911, and it was then decided that the balance of advantage lay with a higher rate of benefit payable from the fourth day of incapacity rather than a lower rate payable from the first day, and that these considerations apply with equal force at present. Serious administrative difficulties would be occasioned if an exception was made in the case of incapacity due to influenza or other infectious diseases. In the final paragraph the Minister states that he is about to introduce into Parliament a Bill to raise the rates of sickness benefit from 10s. to 15s. a week in the case of men and from 7s. 6d. to 12s. in the case of women.

### LIMITED COMPANIES AND EMPLOYERS' SUBSCRIPTIONS.

The Coventry Armaments Output Committee has allotted £1,300 to the Coventry and Warwickshire Hospital out of the surplus funds at its disposal. It will be placed to the Sir Thomas White's Ward Account, in order to secure the gifts contingent upon the total credited to this fund. Mr. W. J. Wormwell favours the Leeds scheme for employers' subscriptions, and has compared some present subscriptions with the amount which would have to be levied in rates if the hospital were run by the municipality. In one case he compared a certain firm's subscription of £50 with a rate on its premises of £166. Most of the Coventry firms are limited companies and not privately owned; but a shareholder, if he is technically an "absentee landlord," is yet a landlord still, and he cannot receive his "rights" without incurring his obligations. The directors, who presumably have local knowledge, can yet regard a subscription to their local hospital as a reasonable duty in gratitude for the work which the hospital does for their men.

### ST. PAUL'S HOSPITAL, ENDELL STREET.

The hospital premises in Endell Street are considerable and have had a somewhat chequered career. There is the vacated building, once known as the "British Lying-in Hospital"; there is also an infirmary. The hospital was translated; the war left neither alone. Now the committee of St. Paul's Hospital has acquired the hospital premises for £15,000. To convert the building the committee needs £40,000, half of which it has borrowed on the security given by its members. Twenty-eight beds are to be provided early; it is expected that the out-patient department will be widely used. But the finances are in a sorry state. Last year the subscriptions were £118 only, and of a statistical income of £2,700 the sum of £1,000 was a grant from the Ministry of Health for "V.D." work. The question of incorporation of some kind is being considered. But the income cannot be left as it is. The first step is to find the right man, if, as seems probable, he has not been found hitherto. Men of conviction are not at a loss for measures. But schemes or appeals are of little use if the man of conviction is wanting.

### IF THE HOSPITALS WOULD COLLECT THEIR DEBTS.

At the Annual Court of Governors of St. George's Hospital, Lord Greville stated that the 1,995 sick and wounded soldiers treated during the war cost the hospital £38,029, but all that was obtained from the Government for the service was £20,715. As we pointed out six months ago (November 8, page 118), the hospitals of London were out of pocket to the extent of about £250,000 for one year alone, by reason of the service they rendered in the matter of the treatment of Service men. If the figures were known it is probable that the voluntary hospitals of the British Isles have an excellent claim on the Government for several million pounds for services rendered. Now the payment of that debt would make all the difference to hospital finance just now, and would tide the hospitals over until better times. It is true that only general hospitals and some special hospitals are concerned, but the general position would be so greatly relieved that King Edward's Hospital Fund and the British Red Cross Society would be in a position to give special grants to hospitals which did not receive the wounded, and consequently have no back debts to collect. The payment of this sum does not need fresh sanction from Parliament, and we offer this as perhaps the most practical suggestion that has been put forward to relieve the present difficult situation.

### ASCOT WEEK AND HOSPITALS.

The Aldershot Hospital and various soldiers' charities are not going to lose the chance offered by Ascot week this year. By order of General Lord Rawlinson, the Aldershot command is to hold a searchlight tattoo on the evenings of June 16, 17, 18, in aid of the Aldershot charities. These dates occur in Ascot week, and it is hoped that those who keep the Ascot festival will attend the tattoo. The charities to benefit from the sale of tickets include the Aldershot Hospital, which, by the way, is on the eve of its twenty-first birthday; the Louise Margaret Hospital Fund for the benefit of soldiers' wives when in hospital; Queen Mary's Home for Soldiers' Children; the Aldershot nursing branch of the Sailors' and Soldiers' Families Association; and the Isolation Hospital Fund. The *Daily Telegraph*, always to the fore in hospital matters, has asked for a thousand pounds to add to the proceeds. We applaud the request because, though entertainments are useful to charity, on charity itself must rest the main ground of appeal if the voluntary system is not to become a mere business in disguise.

### THE PURCHASE OF A PLYMOUTH HOSPITAL.

The committee of the Devon and Cornwall Ear and Throat Hospital, Plymouth, recommends that the present premises should be purchased before the option expires next March. To secure them £1,000 will have to be borrowed, and £300 to be spent on the exterior. The total cost will be about £2,300, towards which, however, half the sum is in hand. Advocating the purchase, Mr. L. R. Dunstan, Mayor of Plymouth, lately made a good point. Referring to the hospital's work and income he said about half the cases were those of school-children, for whom the Education authorities had offered to pay. The Board of Education, however, had declined to sanction the payment; and he gave this as a good instance of the value of "State aid" for voluntary institutions. Whether the reason be "technical" or merely due to red-tape, the fact remains that "efficiency" means merely centralisation, and that a department in Whitehall cannot prevent anomalies of this kind.

### MONEY-LENDERS AND PUBLIC BODIES.

Another amusing incident has occurred in Surrey. Failing, through the effect of official regulations, to borrow in the ordinary way £7,000 for a new diphtheria and scarlet fever block, the Guildford, Godalming, and Woking Joint Hospital Board resorted to the source adopted by private persons in similar difficulty. At the evening meeting the clerk reported that "no applications were received to advertisements asking for terms on which money-lenders would let the Council have the money." The Editor of *Truth* will enjoy the humour of the situation. But it is hard luck on the Council, which is at its wits' end for money.

### PRACTICAL CO-ORDINATION IN LIVERPOOL.

In spite of some subsequent misunderstanding, the details of which need not concern us, an interesting attempt at hospital co-ordination has been made in Liverpool. The recent conference between the hospitals' committee of Liverpool Corporation and the three Poor-Law Boards of the city showed how the Poor Law could assist the corporation by providing beds for cases eligible for the city hospitals. The Select Vestry offered 240 beds at Highfield Hospital for Consumptives, at Knotty Ash; the West Derby Board 800 beds at Belmont Road institution; and the Toxteth Board 150. In the last two institutions any cases can be admitted. Whatever be the issue of this attempt it shows that much can be done by the pooling of the institutional resources of the Poor Law. If the latter can help the voluntary hospitals remains to be seen. But in any case the Ministry of Health has a resource hitherto very much neglected.

### ARE HOSPITALS OVER-STAFFED.

Discussing economy at a recent meeting of the Royal Hampshire County Hospital, the Earl of Northbrook said that it struck him as an outsider that for 151 patients a staff of 110 was rather large. It meant that they had to feed 261 persons daily, which was a consideration when the cost of provisions was so high. He was not able to express an opinion, but it might be well to consider whether they were overstaffed? This raises an important question. What is the correct ratio of staff to patients? Are there available statistics; and is the method of computing the proportion the same?

### THE COST OF SICK VISITING.

An unostentatious Society, of which Lieut.-Col. McAdam Eccles, M.P., is the honorary treasurer, is the Society for the Visitation of the Sick, which appears to be mainly supported by Evangelicals. In 1919 the Society's visitation scheme cost some £425, and the treasurer estimates that each visit, which, of course, may extend to several patients, costs the Society five shillings. The personal service which can be rendered by a properly selected sick visitor is much insisted on by the Society; and perhaps one of the effects of the current financial "scare" in the newspapers may be to encourage volunteers for this and kindred work.

### FEES IN INFECTIOUS HOSPITALS: A PROVOKING POINT.

An interesting point concerning hospitals has been discussed at a meeting of the Bangor City Council, when the Hospital Committee recommended a new scale of charges for patients at the Borough Infectious Hospital. The charges were based on the income of the patients or those responsible for them, and varies from 7s. 6d. a week for incomes up to £100 per annum, to 40s. a week for incomes above £400 a year. The Town Clerk, however, ruled that the proposed charges were illegal. The Council, he said, had no right to differentiate between one ratepayer and another; all must be treated alike. Alderman F. J. Williams, Chairman of the Hospital Committee, said that this differentiation was made in other hospitals in North Wales. They were simply carrying out the income-tax principle. The Town Clerk, however, challenged a member to name a single town in North Wales where such graduated charges were made. The recommendation was referred back to the Committee.

### A HOSPITAL DECLINES A DONATION.

"While giving from a high motive and from a pure source should be encouraged, how far," asks a correspondent, "is an institution justified in sitting in judgment upon the donations it receives?" "A certain hospital," he adds, "has refused to accept the proceeds of a charity football match because it was played on Good Friday." St. Paul's Cathedral once refused a gift of gold plate from a notorious financier. Is a football crowd on Good Friday to be similarly barred? Our own impression is that most of those who support hospitals will regard the action of the committee of the hospital in question as ill-advised; and that a hospital which refuses donations on grounds such as these is likely to regret it.

### THIS WEEK'S DRUG MARKET.

Conditions are much the same as they have been for the past few weeks, and at present there are no indications which point to an improvement in the immediate future. The slow downward tendency of prices is still in evidence, with, however, a tendency here and there in the other direction. Among synthetic drugs phenacetin, amidopyrin, phenazone, resorcin, salol, and barbitone are rather cheaper, and the same may be said of salicylic acid, but sodium salicylate is unchanged. Citric acid has moved downwards in price, but the value of tartaric acid is maintained. There are signs of a downward movement in the value of quinine. The steadier tone of camphor has not been maintained. Cod-liver oil is quiet and unchanged. One of the few articles inclined to be dearer is honey. Sarsaparilla has also been sold at an advanced price. Rhubarb is cheaper, as also are ipecacuanha and balsam of tolu.

